# Associations between housing and management factors and reproductive performance in 327 Norwegian sheep flocks

**DOI:** 10.1186/1751-0147-56-26

**Published:** 2014-05-06

**Authors:** Egil Simensen, Camilla Kielland, Frøydis Hardeng, Knut E Bøe

**Affiliations:** 1 Production Animal Clinical Sciences, Faculty of Veterinary Medicine and Biosciences, Norwegian University of Life Sciences, Campus Adamstuen, Oslo NO-0454, Norway; 2 Animal and Aquatic Sciences, Faculty of Veterinary Medicine and Biosciences, Norwegian University of Life Sciences, Campus Ås, P.O. Box 5003, Oslo NO-1432 Ås, Norway

## Abstract

**Background:**

Housing sheep in insulated, warm buildings, which is common in Norway, involves high buildings costs, but has not been reported to improve health and performance. Few studies have dealt with the association between housing and management and reproductive performance.

Data on housing and management during the indoor period from a questionnaire were merged with herd level data from the Norwegian Sheep Recording System, and the material included 327 sheep flocks. Associations between housing and management factors and reproductive performance were analyzed by means of multivariate regression models and grouped logistic regression.

**Results:**

There was no difference in reproductive performance between warm and cold housing. Number of live born lambs born per pregnant ewe was highest in flocks with 10 or less ewes per pen, and lowest in flocks with more than 40 ewes per pen. Rate of barren ewes was lowest, and number of lambs per ewe at the end of the indoor period was highest in flocks where the sheep were regrouped according to number of foetuses (lambs) identified by scanning. A higher total number of lambs born per pregnant ewe and lambs per ewe at the end of the indoor period were found where other available housing facilities or outdoor areas were used in addition to the main housing unit in the lambing season. The rate of stillbirths was lowest on farms where sheep was the only animal production. None of the factors evaluated had any significant association with mortality of live born lambs in the indoor period. Lowest number of lambs per ewe at the end of the indoor period was found on farms where other family members/hired labour were caretakers as compared with the farmer or spouse/cohabitant, and highest numbers were found where caretakers were older than 60 years of age. Reproductive performance was lower in the Spæl Breed than the Norwegian White Sheep.

**Conclusion:**

Housing systems per se are of minor importance, whereas management practices in the indoor period should be expected to improve reproductive performance.

## Background

In Norway sheep are usually kept indoors in warm, insulated buildings during the winter season [1,2]. However, when comparing warm and cold housing, no differences have been found in the proportion of barren ewes [1], lambs born per pregnant ewe [1,3], neonatal mortality [2], and growth performance of lambs [4]. Moreover, housing sheep in insulated, warm buildings involves high buildings costs [5], without improving reproductive performance.

Still, the variation in reproductive performance is large within Norwegian sheep flocks [6], indicating an association with several other housing and management factors. Experience of the farmer, degree of supervision during lambing, control of colostrum intake, feeding frequency and type of roughage significantly affected neonatal lamb mortality [2]. Pen flooring, ventilation and access to outdoor areas had no effect on this parameter. However, it has been found that access to an outdoor area reduced health problems [7]. Number of lambs born per pregnant ewe was associated with type of roughage and separation of ewe lambs during the winter season [1].

Group size will have an effect on the synchrony of resting and feeding and the time spent queuing in front of the feeding barrier [8], but no data is available showing possible effects on reproductive performance. A large group size will evidently decrease the possibility of feeding the ewes according to their nutritional demand. Regrouping according to the number of foetuses identified by scanning is recommended by the Norwegian Sheep Health Service [9]. Interestingly, grouping of sheep at the start of the indoor period (separation of ewe lambs) was associated with a higher number of lambs born per pregnant ewe [1]. Pen flooring varies a great deal between Norwegian sheep farms [2], but even if it did not affect neonatal mortality, we cannot exclude the possibility that pen flooring can affect other reproductive parameters.

Space allowance is important for the welfare of housed ewes [10]. The sheep barns are usually designed to satisfy the space allowance for the pregnant ewes per see, and the space allowance is often restricted to 0.7 – 0.9 m^2^/ewe [11]. Hence, in the lambing season there is a need for additional space, and many farmers use the feed storage section and even outdoor areas. The supervision during lambing is of great importance [2], but we have limited knowledge as to how the use of additional space may affect reproductive performance.

The purpose of this study was to evaluate possible associations between housing and management factors and the rate of barren ewes, total number of lambs born per pregnant ewe rate of stillbirths, lamb mortality in the indoor period, and lambs alive per ewe at the end of the indoor period. Emphasis was placed on housing and management factors which have not been sufficiently evaluated in previous studies [1,2], i.e. pen design and feeding system, ewes per pen, flooring, grouping, access to outdoor areas and use of housing facilities during the lambing season.

## Material and methods

### Inclusion of herds

A questionnaire regarding housing and management was mailed to the sheep farmers in 2011, and data from the Norwegian Sheep Recording System – NSRS [12] regarding reproductive performance (herd level averages) were used for the same year.

The animal production register of the Norwegian Agricultural Authority includes all 14.000 sheep farmers in Norway entitled to the official subsidy. According to this register one third of the farms with 40–60 winter fed sheep (788 farms), one half with 60–120 sheep (2016 farms), and all farms with more of 120 sheep (2294 farms) was selected. The 5098 farms (flocks) were from all counties of Norway. A letter was sent to the farmers, and they were asked to answer a questionnaire via the Internet (Questback®). A total number of 635 farmers responded (response rate 12.5%), but due to incomplete answers, data from 11 farms were deleted. Thus data from 624 farms were used in the analyses (48 farms with less than 40 sheep, 118 farms with 40–60 sheep, 244 farms with 60–120 sheep and 214 farms with more than 120 sheep).

Out of the 624 farms in the survey, 327 were included in the NSRS. For these farms, data regarding reproductive performance were merged with data from the mail survey, including rate of barren ewes, lambs born per pregnant ewe, mortality of live born lambs in the indoor period, and number of lambs alive per ewe at the end of the indoor period.

### Independent variables

Independent variables from the questionnaire which were included in the analyses of possible associations with the reproductive data from the NSRS were the following: *Descriptive statistics* (flock size and breed), d*emographic measures* (family category, gender and age of caretaker, full time/part time farming, formal agricultural education, sheep husbandry as a part of the total farm production), *housing conditions* (insulated/uninsulated buildings,ventilation, pen design and feeding system, number of sheep per pen, flooring), *management* (grouping of sheep at start of the indoor period and later regrouping, access to outdoor areas, use of housing facilities in the lambing season). The sheep farmers were also asked about space allowance (m^2^/ewe), but the data were obviously not reliable, and were omitted from further analysis. The classification of the independent variables is shown in Tables 1 and 2. Interactions between housing factors in Table 2 using Pearson bivariate correlation are shown in Table 3.

**Table 1:** Distribution of descriptive statistics and demographic measures in the 327 flocks, which were included in the study of potential factors being associated with reproductive performance

**Independent variable**	**Categories**	**No. of flocks**	**% of flocks**
*Descriptive statistics*			
Flock size (number of ewes)^1^	≤40	29	8.9
	41-60	49	15.0
	61-120	133	40.7
	>120	116	35.5
Breed	Norwegian White Sheep	236	72.2
	Spæl Breed	35	10.7
	More than one breed	56	17.1
*Demographic measures*			
Family category of caretaker	Farmer	281	86.7
	Spouse/cohabitant	27	8.3
	Other family members/hired	16	4.9
Gender of caretaker	Female	45	13.8
	Male	282	86.2
Age of caretaker	≤40 years	97	29.7
	41-60 years	183	55.9
	>60 years	47	14.4
Full time/part time farming	Full time farming	157	48.0
	Occupation outside the farm	170	52.0
Formal agricultural education	Yes	197	60.2
	No	130	39.8
Sheep husbandry – total production	Sheep the only production	211	64.7
	Sheep and other animal production	52	16.0
	Other production most important	63	19.3

^1^Flock size was treated as a continuous variable in the analyses.

**Table 2:** Distribution of housing and management factors during the indoor period recorded in the 327 flocks, which were included in the study of potential factors being associated with reproductive performance

**Independent variable**	**Categories**	**No. of flocks**	**% of flocks**
*Housing conditions*			
Housing system	Insulated buildings	221	67.6
	Uninsulated buildings	84	25.7
Ventilation system	Controlled ventilation	197	60.4
	Natural openings	129	39.6
	Mainly outdoors	22	6.7
Pen design and feeding system	Pens with separate feeding table	266	81.3
	Feeding racks inside the pen	61	18.7
Ewes per pen	≤10	78	25.9
	11-20	111	36.9
	21-40	82	27.4
	>40	30	10.0
Flooring	Perforated floors/expanded metal - etc.	255	78.3
	Solid floors, deep litter	51	16.6
	Combinations	20	6.1
*Management*			
Grouping of sheep at start of indoor period	According to age groups	242	74.0
	According to body condition	40	12.2
	No systematic grouping/others	45	13.8
Regrouping during indoor period	Based on body condition	91	27.8
	Based on no. of foetuses (after scanning)	195	59.6
	No particular grouping	41	12.6
Access to outdoor areas	No access	249	76.9
	Access during daytime	36	11.1
	Access all 24 hours	39	12.0
Use of housing facilities during lambing seson	All sheep housed inside the house	87	26.6
	In the sheep house and other available housing facilities	183	56.0
	In the sheep house and outdoors	38	11.6
	Others	19	5.8

**Table 3:** **Interactions between the housing factors in Table **2** in pair wise analyses (Pearson’s correlation coefficient)**

	**Housing system**	**Pen design/feeding system**	**Ewes per pen**	**Flooring**
Housing system	-			
Pen design/feeding system	0.277^**^	-		
Ewes per pen	0.355^**^	0.158^**^	-	
Flooring	0.162^**^	0.163^**^	0.129^*^	-
Access to outdoor areas	0,533^***^	0,401^**^	0,314^**^	0,318^**^

(* = *P <* 0.05, ** = *P <* 0.01, *** = *P <* 0.001).

### Statistical analysis

**Figure 1:**
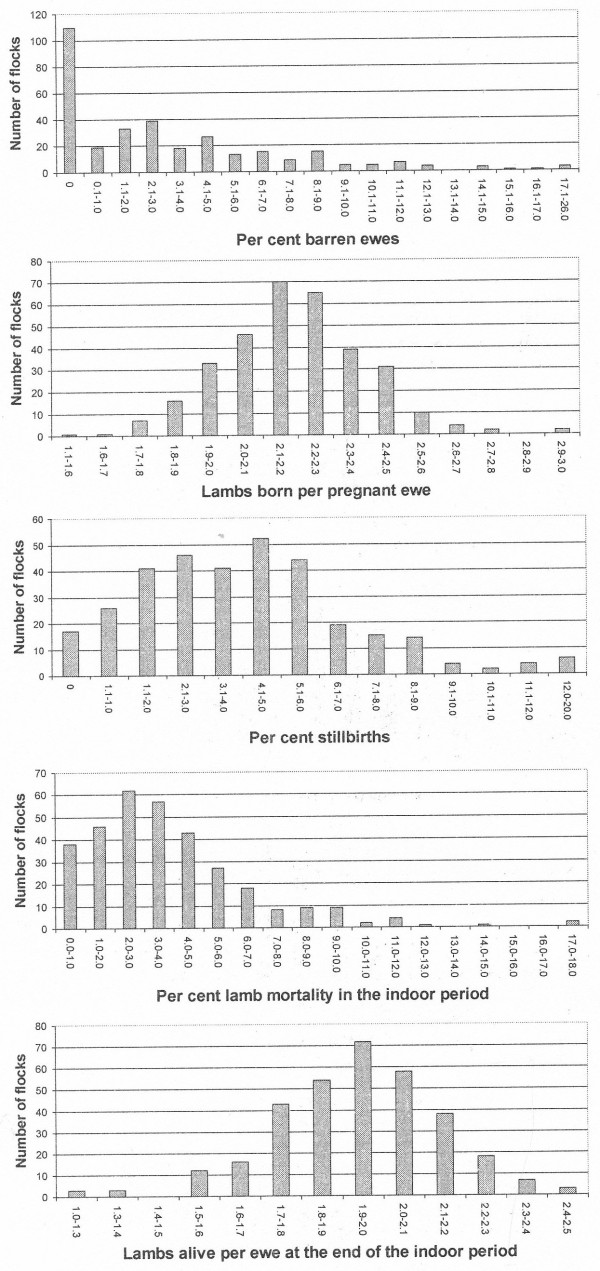
Distribution of the 327 flocks by rate of barren ewes, lambs born per pregnant ewe, mortality rate of live born lambs in the indoor period and lambs alive per ewe at the end of the indoor period.

Dependent variables were the following: Rate of barren ewes, i.e. mated ewes not giving birth to lambs, total number of lambs born (total number, including stillbirths) per pregnant ewe, rate of stillbirths, per cent lamb mortality in the indoor period, and lambs alive per ewe at the end of the indoor period. The distribution of the flocks by these variables is shown in Figure 1. Based on these distributions, associations between the dependent and independent variables were tested in pair wise analyses, i.e. Wilcoxon’s test (rate of barren ewes) and the analyses if variance. Associations with a *P*-value of <0.10 were further tested in multiple regression models. Regarding barren ewes, grouped logistic regression was used after transforming herd data to individual animal level (0–1) based on the rate and flock size, taking into account the herd effect in the analyses. Factors were successively removed from the models in a backward selection until only factors with significant associations (*P <* 0.05) were included. It was guaranteed that there were no interactions between the class variables in the models. Models were assessed using standard techniques for assessing fit and residual patterns.

## Results

### Descriptive statistics and demographic measures

The mean number of ewes per flock was 114.4 (interquartile range 62–138), and the Norwegian White Sheep was the dominant breed (72%). As shown in Table 1, it was mainly the farmer himself, usually a male who was responsible for taking care of the sheep. Approximately 50% of the farming was a part time occupation, and for nearly 65% sheep farming was the only agricultural production at the farm.

### Housing and management

The sheep were kept in insulated (warm) buildings with a controlled ventilation system on approximately 2/3 of the farms (Table 2). The number of ewes per pen varied from 10 or less to more than 40, with 11–20 ewes as the most common group size (40%). The majority of the sheep buildings (nearly 80%) had pens with perforated or slatted flooring. On some of the farms, the ewes had access to an outdoor area during daytime (11.1%) or continuously (12.0%). More than 80% of the sheep buildings had a traditional feeding trough, and the rest had rack or bale feeders inside the pen. There were significant interactions between the housing factors (Table 3). In uninsulated buildings feeding in feeding racks/bale feeders, high number of ewes per pen and deep litter flooring predominated.

At the start of the indoor feeding period, the ewes were grouped according to age on most of the farms, and nearly 60% of the farmers regrouped them after scanning based on number of foetuses (Table 2). There was a significant interaction (*P <* 0.01) between grouping and regrouping. There was also a significant interaction between access to outdoor areas during the indoor period and use of housing facilities in the lambing season (*P <* 0.01).

### Reproductive performance

The mean per cent of barren ewes was 3.31 (interquartile range 0.00-5.00), number of lambs born per pregnant ewe 2.19 (interquartile range 2.04-2.33), rate of stillbirths 4.26 (interquartile range 2.48-6.07), per cent mortality in the indoor period 3.79 (interquartile range 1.96-4.96), and live lambs per ewe at the end of the indoor period 1.94 (interquartile range 1.81-2.07) (Figure 1).

### Results from the statistical models

Table 4 presents an overview of factors being significantly associated with reproductive performance in the models. Of the independent variables included, the following had significant associations with one or more of the reproductive performance parameters: Breed of sheep, family category and age of caretaker, ewes per pen, regrouping during the indoor period, and use of housing facilities during the lambing season. The results are shown in Tables 5, 6, 7 and 8 and can be summarized as follows:

**Table 4:** Results from the statistical models where significant associations were found between herd factors and reproductive performance

	**Barren ewes, %**	**Lambs born per pregnant ewe**	**Stillbirths,%**	**Lamb mortality,%**^**a**^	**Lambs per ewe**^**b**^
*Descriptive statistics*					
Breed	*	***			**
*Demographic measures*					
Family category of caretaker					*
Age of caretaker					*
Sheep husbandry – total production			*		
*Housing and management*					
Ewes per pen		*			
Regrouping during the indoor period	**				***
Use of housing facilities during the lambing season		**			**

(*P = <0.05, **P = <0.01, ***P = 0.001).

^a^Mortality rate of live born lambs in the indoor period.

^b^Lambs per ewe at the end of the indoor period.

**Table 5:** **Results from the analysis of factors being significantly associated with barren ewes**^**1**^

	**n**	**OR**	**95 CI of OR**	***P***
*Breed*				
Norwegian White Sheep and other heavy breeds	236	1.00	-	
Spæl and other light breeds	35	1.14	0.94-1.38	0.199
More than one	56	1.32	1.16-1.50	0.002
*Regrouping during the indoor period*				
Based on body condition	91	1.00	-	
Based on no. of foetuses (after scanning)	195	0.59	0.52-0.67	<0.001
No particular grouping	41	1.31	1.10-1.55	0.002

Number of flocks (n), odds ratio (OR) and 95% confidence interval (CI) adjusted for flock size.

^1^Mated ewes not giving birth to lambs.

**Table 6:** Results from the analyses of housing and management factors being significantly associated with total number of lambs born per pregnant ewe

	**Lambs born per pregnant ewe**			
	**Number of flocks**	**Mean**	**SE**	***P***
*Breed*				
Norwegian White Sheep and other heavy breeds	236	2.19	0.02	a
Spæl and other light breeds	35	2.09	0.04	b
More than one breed	56	2.08	0.03	b
*Ewes per pen*				
≤10	78	2.18	0.03	a
11-20	111	2.12	0.02	b
21-40	82	2.11	0.03	b
>40	30	2.06	0.04	b
*Use of housing facilities during the lambing season*				
All sheep housed inside the sheep house	87	2.09	0.03	ac
In the sheep house and other available housing facilities	183	2.16	0.02	bd
In the sheep house and outdoors	36	2.20	0.04	bd
Others	19	2.02	0.05	ac

Different letters in the probability (*P*) column indicate significant differences (*P <* 0.05).

**Table 7:** Results from the analyses of housing and management factors being significantly associated with rate of stillbirths

	**Lambs born per pregnant ewe**			
	**Number of flocks**	**Mean**	**SE**	***P***
*Sheep husbandry – total production*				
Sheep the only production	211	4.29	0.21	a
Sheep and other animal production	52	5.29	0.42	b
Other production most important	63	5.21	0-38	b

Different letters in the probability (*P*) column indicate significant differences (*P* < 0.05).

**Table 8:** Results from the analyses of housing and management factors being significantly associated lambs per ewe at the end of the indoor period

	**Lambs per ewe**			
	**Number of flocks**	**Mean**	**SE**	***P***
*Breed*				
Norwegian White Sheep	236	1.90	0.02	a
Spæl Breed	35	1.82	0.04	b
More than one breed	56	1.81	0.03	b
*Family category of caretaker*				
Farmer	281	1.87	0.02	a
Spouse/cohabitant	27	1.91	0.04	a
Other family members/hired	16	1.74	0.05	b
*Age of caretaker*				
≤40 years	97	1.81	0.03	a
41–60 years	183	1.83	0.03	b
>60 years	47	1.89	0.03	b
*Regrouping during the indoor period*				
Based on body condition	91	1.83	0.03	ac
Based on no. of foetuses (after scanning)	195	1.90	0.03	b
No particular grouping	41	1.81	0.04	ac
*Housing during the lambing season*				
All sheep housed inside the house	87	1.84	0.03	acd
In the sheep house and other available housing facilities	183	1.88	0.03	ad
In the sheep house and outdoors	36	1.91	0.04	acd
Others	19	1.74	0.05	b

Different letters in the probability (*P*) column indicate significant differences (*P* < 0.05).

#### Descriptive statistics

Flock size: No significant associations were found between flock size and any of the performance parameters.

Breed: The Spæl Breed had a higher rate of barren ewes, lower number of lambs born per pregnant ewe and lambs per ewe at the end of the indoor period compared with the Norwegian White Sheep.

#### Demographic measures

Family category of caretaker: Lowest number of lambs per ewe at the end of the indoor period was found on farms were other family members/hired labour were caretakers as compared with the farmer or spouse/cohabitant.

Age of caretaker: Highest number of lambs per ewe at the end of the indoor period was found in flocks where caretakers were older than 60 years of age.

Sheep husbandry – total production: The rate of stillbirths was lowest on farms where sheep was the only animal production.

#### Housing conditions and management

Ewes per pen: Number of live lambs born per pregnant ewe was highest in flocks with 10 or less ewes per pen, and lowest in flocks with more than 40 ewes per pen.

Regrouping during the indoor period: Rate of barren ewes was lowest in flocks where the sheep were regrouped according to number of lambs (foetuses) identified by scanning. (Regrouping was significantly associated with grouping at the start of the indoor period). Regrouping was also significantly associated with a higher number of lambs per ewe at the end of the indoor period.

Use of housing facilities during the lambing season: There was a higher total number of lambs born per pregnant ewe, and lambs per ewe at the end of the indoor period in flocks where other available housing facilities or outdoor areas were used in addition to the main housing unit.

None of the factors evaluated in the models had any significant association with mortality of lambs born alive in the indoor period.

## Discussion

When selecting sheep farms for this study the emphasis was placed on the larger and more professional sheep farmers in Norway as reflected in the large mean herd size in the final dataset. The fact that the questionnaire was distributed by mail, no reminder was sent, and the farmers were asked to respond via Internet explains the low response rate. Average flock size was 114.4 ewes, vs 71.6 for all sheep farms in Norway (76.1 for farms attending NSRS), but the reproductive performance data were representative for all flocks attending NSRS (number of lambs born per mated ewe 2.11 vs 2.07, stillbirth rate 4.62 vs 4.51, lamb mortality in the indoor period 3.79 vs 3.60).

The results from the present study confirm previous results [1-3] in that there was no difference in performance between warm and cold housing. The only housing factor which was significantly associated with performance was the number of ewes per pen, i.e. highest total number of lambs born per pregnant ewe in pens with 10 or fewer ewes and lowest in pens with more than 40 ewes. A possible explanation may be better control and follow up around the time of mating and during the pregnancy period when the sheep are kept in smaller groups. It may also be related to behavioral traits, as sheep in larger group sizes show decreased synchrony in resting and feeding behavior and reduced the time spent queuing in front of the feeding barrier [8].

Two of the housing factors studied, pen design/feeding system and flooring, did not have any significant association with reproductive performance. Another factor, space allowance, is an important factor in relation to sheep welfare [10]. Unfortunately, this factor could not be included in the analyses due to lack of reliable data.

Two of the management factors, regrouping and use of housing facilities in the lambing season may possibly be related to flock size. Systematic regrouping and use of other available rooms or outdoor areas during the lambing season is more important in the larger flocks, and flock size may thus be a confounding factor. However, flock size was not significantly associated with any of the reproductive performance parameters in the pair wise analyses.

In the pair wise analyses, regrouping of ewes had a stronger association with the rate of barren ewes than grouping at the start of the indoor period, and was therefore used in the model, even if regrouping was undertaken after the mating season. Regrouping was associated with a lower rate of barren ewes and a higher number of lambs per ewe at the end of the indoor period. Grouping/regrouping makes it possible to provide better feeding and care according to the needs of the sheep. However, systematic grouping and regrouping may also be an indication of generally better management in flocks with this practice. In a previous study [1] separation of ewe lambs was associated with a higher number of lambs born per pregnant ewe and a higher autumn live weight of the lambs.

Keeping sheep outdoors or in other available housing facilities during the lambing season in addition to the main building unit was associated with a higher number of lambs born per pregnant ewe and lambs per ewe at the end of the indoor period. These practices were significantly correlated with access to outdoor areas during the whole indoor period, which may have a positive influence on reproduction. Use of extra areas around and after lambing may also reduce the risk of lamb mortality, as less health problems were found in flocks where the sheep had access to outdoor areas, particularly during the lambing season [7]. However, no associations between housing/management and lamb mortality were found in the present study.

Demographic measures were included in the analyses to take into account associations related to housing and management which otherwise could not be explained. It was found that both family category and age of caretaker were significantly associated with number of lambs per ewe at the end of the indoor period. The number of lambs was lower on farms where caretakers were other family members/hired labour compared with the farmer or spouse/cohabitant and highest on farms where caretakers were older than 60 years of age. In another study it was found that rate of barren ewes was highest on farms with sheep only, run by the younger farmers, and lowest on farms with other livestock enterprises (mainly dairy cattle) and run by the older farmers [1]. A higher rate of neonatal survival was found where the farmer had at least 15 years of experience in sheep farming [2]. These associations may be related to experience with sheep farming. The rate of stillbirths was lowest on farms where sheep was the only animal production. This indicates that farmers with other animal productions in combination with sheep were less dedicated to sheep farming.

The Spæl Breed had a significantly lower reproductive performance than the Norwegian White Sheep (higher rate of barren ewes, lower number of lambs born per pregnant ewe, and lambs alive per ewe at the end of the indoor period). This is in agreement with previous results [1]. Flocks in which the Spæl Breed predominated had a lower risk of neonatal deaths compared to flocks where the Norwegian White Sheep predominated [2].

## Conclusion

Housing systems per se were found to be of minor importance, whereas management practices in the indoor period should be expected to improve reproductive performance.

## Competing interests

All authors declare that they have no competing interests.

## Authors’ contribution

ES and CK carried out the mail survey, FH organized the NSRS data as basis for statistical analyses, and KB assisted in preparing the manuscript. ES was the head writer of the manuscript. All authors have read and approved the final manuscript.
